# A novel pathogenic variant of *DNMT3A* associated with craniosynostosis: a case report of Heyn–Sproul–Jackson syndrome

**DOI:** 10.3389/fped.2023.1165638

**Published:** 2023-05-25

**Authors:** Ga Hye Kim, Jaewon Kim, Jaewoong Lee, Dae-Hyun Jang

**Affiliations:** ^1^ Department of Rehabilitation Medicine, Incheon St. Mary's Hospital, College of Medicine, The Catholic University of Korea, Seoul, Republic of Korea; ^2^ Department of Laboratory Medicine, Incheon St. Mary's Hospital, College of Medicine, The Catholic University of Korea, Seoul, Republic of Korea

**Keywords:** Heyn-Sproul-Jackson syndrome, DNMT3A, craniosynostosis, novel variant, microcephaly

## Abstract

Pathogenic variants of *DNMT3A* have been implicated in Tatton-Brown-Rahman syndrome, an overgrowth disorder with macrocephaly and intellectual disability. However, there are recent reports of variants in the same gene giving rise to an opposing clinical phenotype presenting with microcephaly, growth failure, and impaired development—named Heyn-Sproul-Jackson syndrome (HESJAS). Here, we present a case of HESJAS caused by a novel pathogenic variant of *DNMT3A*. A five-year-old girl presented with severe developmental delay. Perinatal and family history were non-contributory. Physical exam showed microcephaly and facial dysmorphic features, and neurodevelopmental assessments revealed profound global developmental delay. Brain magnetic resonance imaging findings were normal; however, brain 3D computed tomography revealed craniosynostosis. Next generation sequencing revealed a novel heterozygous variant in *DNMT3A* (NM_175629.2: c.1012_1014 + 3del). The patient's parents did not carry the variant. In this report, a novel feature associated with HESJAS (craniosynostosis) is described, along with a more detailed account of clinical manifestations than those in the original report.

## 1. Introduction

Heyn–Sproul–Jackson syndrome (HESJAS, OMIM #618724) was first described in 2019 by Heyn et al. as a novel cause of microcephalic dwarfism (1). The authors reported three unrelated patients, each aged 13 years, 19 months, and 4.5 years with microcephaly, short stature, low weight, and global developmental delay. HESJAS is caused by heterozygous pathogenic variants in *DNMT3A* [DNA (cytosine-5)-methyltransferase 3A] on chromosome 2p23. *DNMT3A* codes for a DNA methyltransferase responsible for DNA methylation patterns in mammals, one of the major epigenetic mechanisms influencing gene expression and cell differentiation (2). Heterozygous variants in the same gene can also cause Tatton-Brown-Rahman syndrome (TBRS, OMIM #615879), a reciprocal phenotype presenting with macrocephaly, overgrowth, and intellectual disability (3). To date, more than 90 individuals with a pathogenic variant in *DNMT3A* related to TBRS have been reported (4); however, there have been no cases of HESJAS since the initial report. Herein, we describe a patient carrying a novel heterozygous *DNMT3A* variant, c.1012_1014 + 3del, identified by next generation sequencing, who showed clinical features of HESJAS.

## 2. Case presentation

### 2.1. Clinical presentation

The patient was referred to a center for genetic and rare diseases at age 5 with severe developmental delay. She was delivered by cesarean section at 37 weeks of gestation with a birth weight of 2.5 kg (−0.7 SD) and no known perinatal complications. She was the third child of nonconsanguineous parents, and her two older siblings had no developmental problems. Her growth parameters at the time of referral were as follows: weight 17 kg (−1.3 SD) and head circumference 48 cm (−2.3 SD). When we attempted to take a height measurement, the patient was fearful of the digital height scale and became extremely agitated and fitful. Thus, we could not take an accurate measurement of her height, and her parents also stated that she had never had her height measured after birth. The parents estimated her height to be about average compared to peers of the same age, although the parents and her siblings are taller compared to the average population. Her body proportion was proportionate, and skeletal x-ray films showed no abnormalities. Physical examination revealed facial dysmorphic features. She had a prominent epicanthus, hypertelorism, mildly down-slanted palpebral fissures, a broad nasal tip, and a depressed nasal bridge. She started walking independently at 36 months of age.

Various neurodevelopmental assessments were performed. The Korean version of the Vineland Social Maturity Scale (K-SMS) revealed an SQ of 47.1. Her Childhood Autism Rating Scale (CARS) score was 27. Her Beery Visual-Motor Integration-6 (VMI-6) score was 50, corresponding to an equivalent age of 2 years and 8 months. On the Bayley Scales of Infant and Toddler Development (3rd edition) done at age 5 years, her development corresponded to an equivalent age of 26 months in cognition, 19 months in receptive language, 17 months in expressive language, 34 months in fine motor skills, and 24 months in gross motor skills. Overall, she displayed profound global developmental delay.

**Figure 1:**
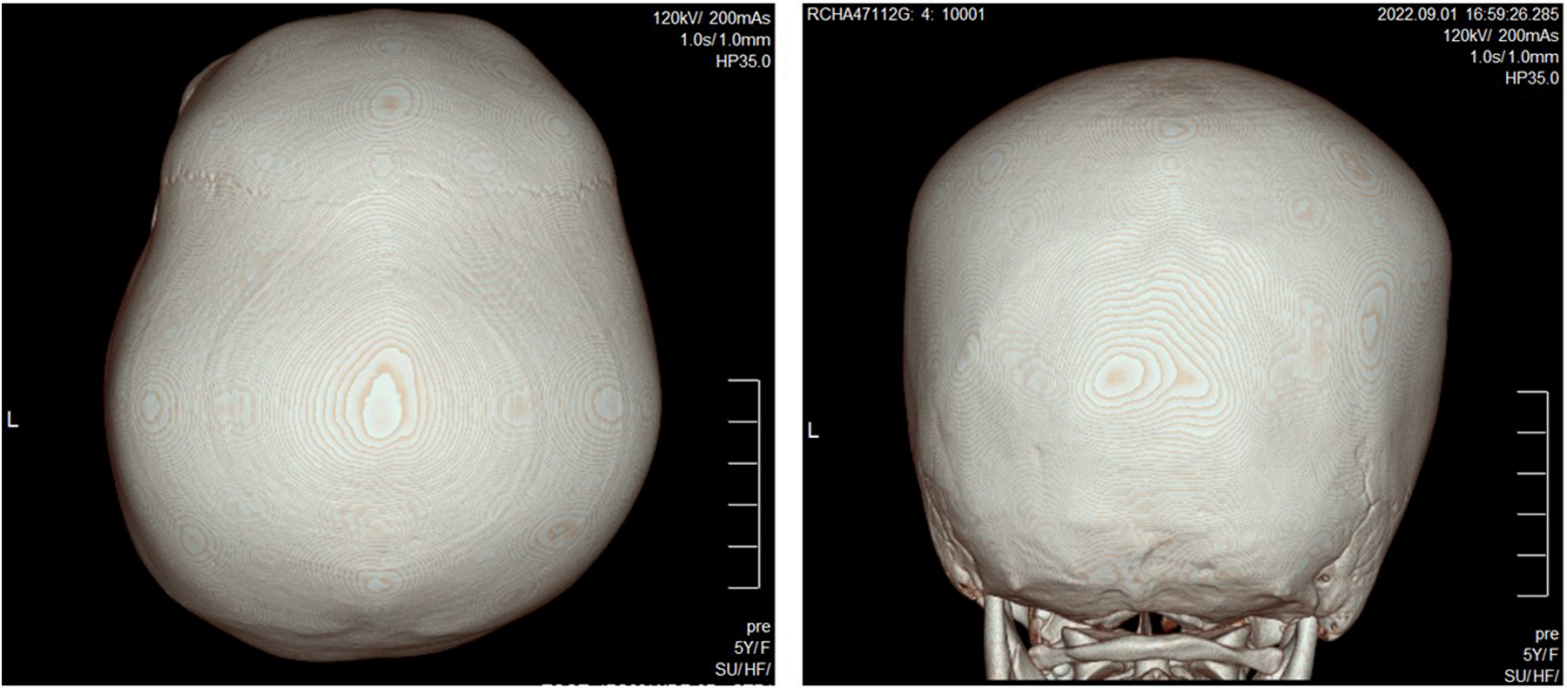
3D CT reconstruction of the skull shows closed sagittal and bilateral lambdoid sutures.

Laboratory tests, including a complete blood count with cell morphology, blood chemistry, electrolytes, and thyroid function revealed parameters within normal ranges. The results of serum amino acid, urine organic acid, and plasma acylcarnitine tests to rule out metabolic disorders were also within normal range. Brain magnetic resonance imaging (MRI) was normal; however, brain 3D computed tomography (CT) revealed craniosynostosis (sagittal and bilateral lambdoid suture fusion state) (Figure 1).

### 2.2. Cytogenetic and molecular analyses

Chromosomal studies revealed a karyotype of 46, XX without anomalies. No significant microdeletions or duplications were detected via chromosomal microarray testing. The methylation specific polymerase chain reaction-restriction fragment length polymorphism (PCR-RFLP) technique for Angelman Syndrome showed no abnormalities.

**Figure 2:**
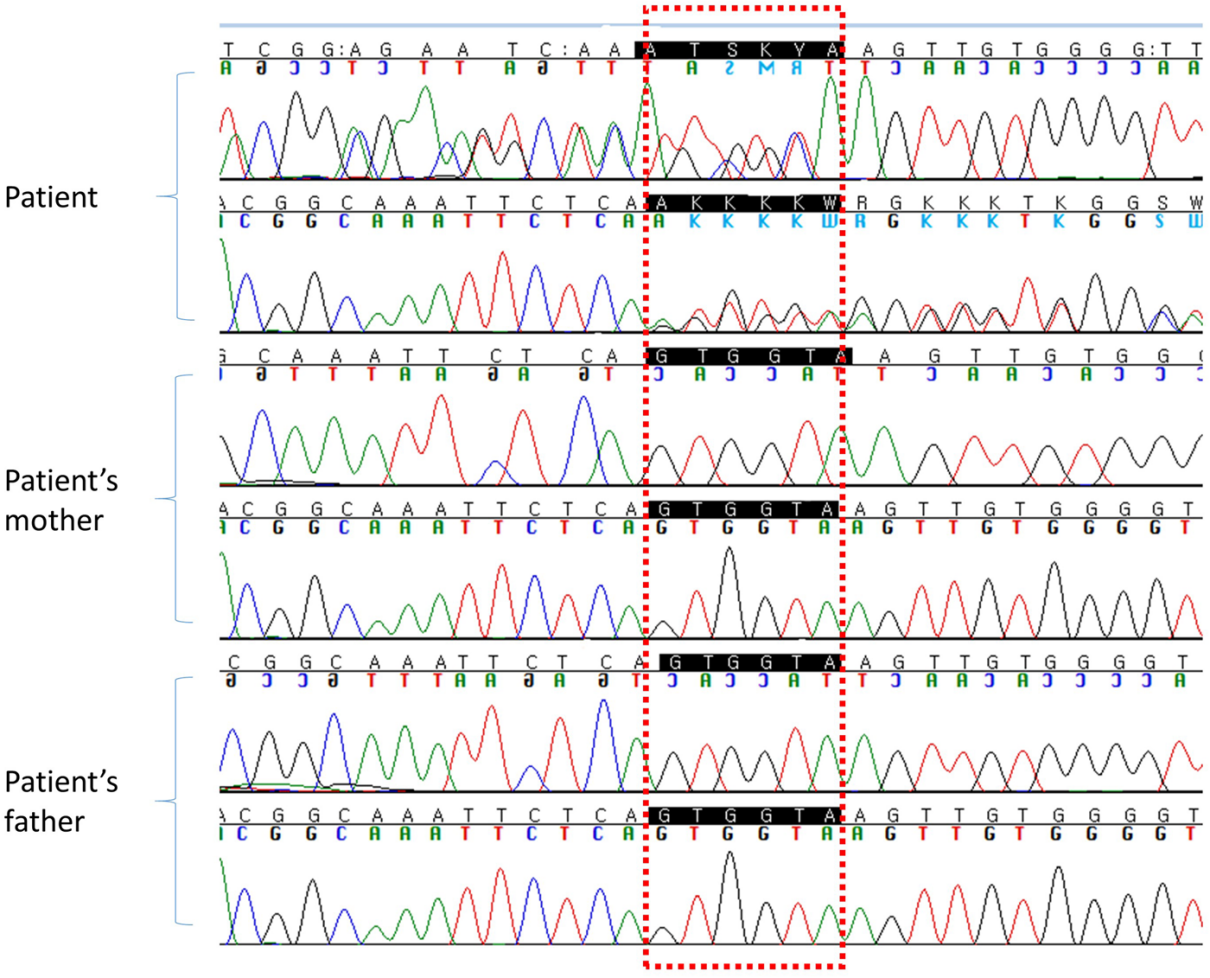
DNA sequencing chromatogram for the patient and her parents.

Next generation sequencing analysis of a multi-gene panel consisting of 985 genes related to delayed development was performed. Libraries were prepared using the Illumina Nextera DNA kit, and sequencing was performed using the TruSight One Sequencing Panel Kit (Illumina Inc., San Diego, CA, USA). A heterozygous variant was identified in exon 8 of *DNMT3A* (NM_175629.2: c.1012_1014 + 3del). This variant was classified as *de novo* compared with the results obtained in the conventional Sanger sequencing of the parents (Figure 2).

Using the SpliceAI program (https://spliceailookup.broadinstitute.org/), an in silico splicing prediction tool, the variant was predicted to lead to loss of splice donor site (score 0.94). This variant possibly leads to exon 8 skipping, consistent with an in-frame deletion of 53 amino acids [r.(856_1014del), p.(286_338del)] involving the PWWP domain. Although it is impossible to confirm without functional testing, previous reports of HESJAS involving the PWWP domain (1), in conjunction with the patient's clinical phenotype consistent with HESJAS likely means that the variant may also be gain-of-function. There have been cases of in-frame deletions leading to truncated forms of proteins, which behave as gain-of-function variants (5). This variant has not previously been reported in large-scale population databases (gnomAD and EXAC)*.* It is classified to be “likely pathogenic” based on the American College of Medical Genetics and Genomics guidelines regarding the interpretation of sequence variations (PS2 + PM2 + PM4 + PP4) (6).

## 3. Discussion

Herein, we describe a case of HESJAS caused by a pathogenic variant in *DNMT3A*. To our knowledge, this is the first reported case of HESJAS since the initial report. Although *DNMT3A* variants have been associated with an overgrowth syndrome, the major clinical features of TBRS are overgrowth (either in tall stature or macrocephaly, 2 SDs above population mean) and intellectual disability. When these two features are present and a constitutive variant of *DNMT3A* is found, TBRS diagnosis is likely (7). However, given our patient's head circumference of below 2 SDs and low weight, our patient verges on the side of undergrowth, more consistent with HESJAS. The patient showed profound global developmental delay and exhibited microcephaly and facial dysmorphism. Weight was within −2 SDs. Brain MR findings were normal, and NGS analysis identified a novel heterozygous variant in *DNMT3A* (c.1012_1014 + 3del). While our patient shares some characteristics with the previously reported patients (microcephaly and intellectual disability), an entirely new feature, craniosynostosis was discovered in our patient.

In 2019, Heyn et al. identified three patients with two novel variants (c.988T > C, c.997G > A) of *DNMT3A* who showed clinical manifestations of growth restriction, microcephaly, and impaired intellectual impairment (1). All three patients showed prenatal growth restriction, which became more evident postnatally (height and weight both below 2 SD). All three patients also had microcephaly and displayed some sort of malformation or dysmorphic features (Table 1). Moderate-to-severe developmental delay was noted in all three patients, so much so that one patient showed no evident speech at age 13. The detailed developmental progress of the other two patients was not given. As for our patient, her parents did not report any particular prenatal growth restriction, and her birth weight (2.5 kg) was just at the cusp of the low birth weight (LBW) range. At age 5 when she was referred, her weight was within 2 SDs, however she showed definite microcephaly (head circumference 48 cm, −2.31 SD). It is possible that her weight may have been more influenced by nutrition, exercise, and other environmental factors; thus, our patient showed less severe growth restriction than previously reported HESJAS patients. Facial dysmorphic features were noted as above in our patient without significant malformations of the limbs. Consistent with previous reports, our patient had severe global developmental delay with minimal expressive speech at age 5. A novel characteristic in our patient was the existence of craniosynostosis. Clinical characteristics of previous patients and our patient are summarized in Table 1.

**Table 1:** Summary of variants and clinical findings in individuals with *DNMT3A*-related HESJAS.

Patient (source of study) Country of origin	Proband, this study South Korea	P1 (1) USA	P2 (1) New Zealand	P3 (1) Spain
Variant	c.1012_1014 + 3del (*de novo*)	c.988T > C (*de novo*)	c.988T > C (*de novo*)	c.997G > A (*de novo*)
Gender	Female	Female	Male	Male
Age at measurement	5 years 7 months	1 year	1 year 7 months	4 years 6 months
Birth weight	2.5 kg (−0.7 SD)	1.9 kg (−2.8 SD)	2.3 kg (−1.1 SD)	1.9 kg (−2.3 SD)
Height	Uncheckable^†^	60.5 cm (−5.4 SD)	68.9 cm (−4.6 SD)	93.8 cm (−3.2 SD)
Weight	17 kg (−1.3 SD)	5.79 kg (−4.7 SD)	6.64 kg (−5.2 SD)	11 kg (−2.6 SD)
Head circumference	48 cm (−2.3 SD)	41.5 cm (−4.1 SD)	41 cm (−6.6 SD)	NR (microcephaly)
Craniosynostosis	+	NR*	NR	NR
Neurocognitive impairment	Severe	Severe	Moderate	Moderate-severe
	Minimal expressive speech at age 5			
Dysmorphic features	Prominent epicanthus, hypertelorism, broad nasal tip, depressed nasal bridge	Sparse hair 11 pairs of ribs	Short broad metacarpals and phalanges	Strabismus, epicanthic folds, wide forehead, sparse hair; short broad metacarpals and phalanges; bilateral macro-orchidism
Brain MRI	Normal	NR	Normal	Normal

* NR, not reported.

^†^ Height measurement was not possible due to the patient's fear of the height scale. She was assumed to be about average in height compared to same age peers.

*DNMT3A*, along with *DNMT3B*, encodes for one of two enzymes responsible for *de novo* DNA methylation, whereas a third methyltransferase, DNMT1, accounts for maintenance DNA methylation during active replication. *DNMT3A*, in addition to CpG methylation, also partially accounts for non-CpG methylation in embryonal stem cells and neural cells (8, 9). Non-CpG methylation was reported to correlate with transcriptional repression (10) and the pluripotency-associated epigenetic state (11). Other enzymes also modify the protein component of chromatin, known as histones. Together these modifications act as epigenetic regulators of gene expression.

The role of *DNMT3A* in physiological growth was first identified by an array of patients exhibiting overgrowth syndrome with *de novo* germline missense and truncating loss-of-function variants (3, 12). However, Heyn et al. reported a set of patients with a contrasting phenotype of reduced growth involving variants of that very same gene. They posited that since *DNMT3A* haploinsufficiency leads to overgrowth (13), this suggests that the pathogenic variants in their patients were genetic gain-of-function variants. They found that in individuals with the newly discovered *DNMT3A* gain-of-function variants, key developmental genes were hypermethylated relative to controls. The pattern of histone modifications at these promoters was also altered. Specifically, the hypermethylation of Polycomb-associated differentially methylated regions (DMRs) and DNA methylation valleys (DMVs) led to a secondary reduction in the histone H3K27me3 due to impaired binding of Polycomb repressive complex 2 (PRC2) to methylated DNA (1).

In relation to H3K27me3 modification, a notable feature of our patient is the presence of craniosynostosis. 3D CT imaging revealed sagittal and bilateral lambdoid suture fusion in our proband. Given the patient's microcephaly and synostosis, we considered surgical treatment. However, surgical reconstruction for craniosynostosis is generally performed within the first year of life (14). Our patient was already 5 years old when she visited our clinic for the first time, and global developmental delay was severe. After much consideration, we decided against surgical measures and elected to monitor the state of her coronoid sutures during follow-up visits. Craniosynostosis occurs due to the premature ossification of single or multiple skull sutures, resulting in skull deformities. Although single gene mutations are known to cause craniosynostosis in many cases, recently an epigenetic basis for the development of craniosynostosis has been suggested (15, 16). Studies of monozygotic twins where only one of the twin pair had craniosynostosis suggest epigenetics at play in non-syndromic craniosynostosis (17, 18). Several studies have reported monozygotic twins developing variable phenotypes and severities with regard to craniosynostosis (18, 19). One study discovered DNA methylation and histone acetylation in one-third of monozygotic twin pairs with incongruous presentations (20). Taken together, these results suggest epigenetic involvement in craniosynostosis formation.

Previous studies have identified histone H3K27me3 as an important gene repressor involved in osteogenic commitment and skeletal development. Enhancer of Zeste Homolog 2 (EZH2), a member of PRC2, is an enzyme that catalyzes the trimethylation of H3K27me3. EZH2 is critical in preserving the pluripotency of hematopoietic, muscle, and neural stem cells (21). EZH2 is also involved in tissue-specific differentiation, including stimulating adipogenic differentiation (22) and inhibiting osteogenic differentiation (23). Dudakovic et al. (24) demonstrated *in vivo* that blocking EZH2 and in turn, lowering H3K27me3 activity, stimulated osteogenic differentiation while suppressing adipogenic differentiation. Heyn et al. in their original report of HESJAS, showed that H3K27me3 levels were reduced in patients' fibroblasts but levels of EZH2 were normal, indicating that *DNMT3A*-mediated DNA methylation which directly inhibits PRC2 binding and activity, probably leads to a secondary reduction in H3K27me3 (1). Nonetheless, the final result of reduced H3K27me3 may play a role in premature ossification, which perhaps provides an explanation for the craniosynostosis we observed in our patient. Whether or not craniosynostosis was tested for in the three patients previously reported to have HESJAS was not mentioned in the original study.

Previously identified genes associated with microcephalic dwarfism disrupt cell proliferation to reduce cell number and organism size (25); however, Heyn et al. in their original study observed “a transcriptional bias away from pluripotency toward differentiation” in mouse neural progenitor cells containing their patient's orthologous substitution (1). Therefore, they suggest that gain-of-function *DNMT3A* pathogenic variants may instead increase cellular differentiation causing premature depletion of progenitor cells, and decrease final cell numbers in tissues and consequently organism size. Further evaluation of our patient's variant is warranted to understand its exact and specific mechanism. In conclusion, we report a novel pathogenic variant of *DNMT3A* corresponding to the rarely reported HESJAS, and this case report is distinguished from previous reports in that we give a more detailed clinical picture including developmental evaluations of the patient.

## Data Availability

The datasets presented in this study can be found in online repositories. The names of the repository/repositories and accession number(s) can be found below: ClinVar SUB13004300 (SCV003845958).
